# VNP20009-Abvec-Igκ-MIIP suppresses ovarian cancer progression by modulating Ras/MEK/ERK signaling pathway

**DOI:** 10.1007/s00253-024-13047-z

**Published:** 2024-02-19

**Authors:** Qian Wang, Yuwen Tang, Ang Dai, Tiange Li, Yulin Pei, Zuo Zhang, Xinyue Hu, Tingtao Chen, Qi Chen

**Affiliations:** 1 Department of Obstetrics and Gynecology, The 2nd Affiliated Hospital, Jiangxi Medical College, Nanchang University, 1 Minde Road, Donghu District, Nanchang City, 330000 Jiangxi Province China; 2 National Engineering Research Center for Bioengineering Drugs and the Technologies, Institute of Translational Medicine, Jiangxi Medical College, Nanchang University, No. 1299, Xuefu Avenue, Honggutan District, Nanchang City, Jiangxi Province China

**Keywords:** VNP20009, MIIP, Bacterial therapy, Engineered bacteria, Ovarian cancer

## Abstract

**Abstract:**

Ovarian cancer poses a significant threat to women’s health, with conventional treatment methods encountering numerous limitations, and the emerging engineered bacterial anti-tumor strategies offer newfound hope for ovarian cancer treatment. In this study, we constructed the VNP20009-Abvec-Igκ-MIIP (VM) engineered strain and conducted initial assessments of its in vitro growth performance and the expression capability of migration/invasion inhibitory protein (MIIP). Subsequently, ID8 ovarian cancer cells and mouse cancer models were conducted to investigate the impact of VM on ovarian cancer. Our results revealed that the VM strain demonstrated superior growth performance, successfully invaded ID8 ovarian cancer cells, and expressed MIIP, consequently suppressing cell proliferation and migration. Moreover, VM specifically targeted tumor sites and expressed MIIP which further reduced the tumor volume of ovarian cancer mice (*p* < 0.01), via the downregulation of epidermal growth factor receptor (EGFR), Ras, p-MEK, and p-ERK. The downregulation of the PI3K/AKT signaling pathway and the decrease in Bcl-2/Bax levels also indicated VM’s apoptotic potency on ovarian cancer cells. In summary, our research demonstrated that VM exhibits promising anti-tumor effects both in vitro and in vivo, underscoring its potential for clinical treatment of ovarian cancer.

**Key points:**

• *This study has constructed an engineered strain of Salmonella typhimurium capable of expressing anticancer proteins*

• *The engineered bacteria can target and colonize tumor sites in vivo*

• *VM can inhibit the proliferation, migration, and invasion of ovarian cancer cells*

**Graphical Abstract:**

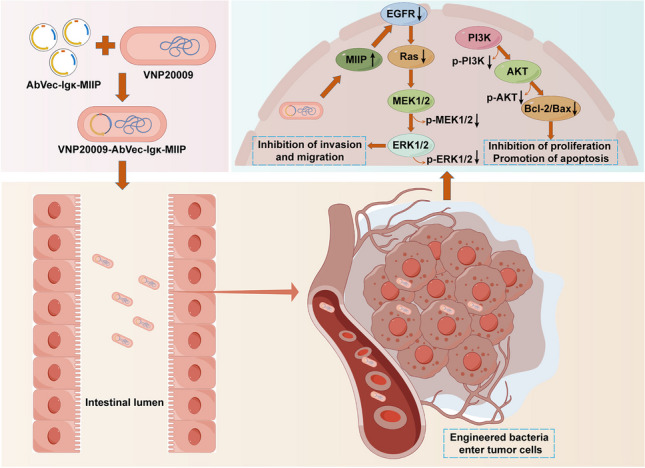

**Supplementary Information:**

The online version contains supplementary material available at 10.1007/s00253-024-13047-z.

## Introduction

Ovarian cancer ranks as the third most prevalent gynecological malignancy globally, boasting the unfortunate distinction of having the highest fatality rate (Kuroki and Guntupalli 2020). Its 5-year survival rate is approximately 48% (Siegel et al. 2021). Owing to the subtle clinical symptoms in the initial phases, advanced stages of ovarian cancer are diagnosed in over 70% of patients (Schoutrop et al. 2022). These advanced stages are marked by the extensive spread of cancer to the pelvic and abdominal regions, accompanied by the accumulation of ascites, ultimately resulting in rapid disease progression (Nasioudis et al. 2018). Furthermore, recurrence and metastasis constitute the primary causes of patient mortality (Yamada et al. 2021). Currently, conventional treatment modalities represented by chemotherapy and targeted drugs exhibit challenges such as strong toxic side effects, low specificity, and susceptibility to drug resistance (Gujrati et al. 2014). Therefore, pursuing a novel approach capable of effectively restraining the advancement of ovarian cancer holds significant importance in clinical treatment.

Currently, the precise etiology and pathogenesis of ovarian cancer remain incompletely understood, possibly involving molecular genetic and genomic alterations, including point mutations, gene amplifications, deletions, and translocations (Cho and Shih Ie 2009). In recent years, numerous genetic loci related to ovarian cancer, including BRCA1/2, TP53, PTEN (Kang et al. 2020), and associated protein molecules such as CA125, HE4, and AFP (Matsas et al. 2023), have become research focal points. These have been found to potentially correlate with the clinical characteristics, recurrence, metastasis, and other biological attributes of ovarian cancer (Gusev et al. 2019). Hence, identifying particular genes related to the aggressive nature of ovarian cancer and investigating potential mechanisms can significantly enhance the effectiveness of precision therapies. Migration and Invasion Inhibitory Protein (MIIP) is a newly discovered tumor-suppressive gene in recent years. It is located in the Ip36.22 region of the chromosome and encodes a highly hydrophilic protein composed of 388 amino acids (Song et al. 2003). Previous research findings have indicated the downregulation of MIIP in a range of malignancies, encompassing endometrial cancer, ovarian cancer, breast cancer, prostate cancer, non-small cell lung cancer, glioma, and colon cancer (Du and Wang 2019; Hu et al. 2020; Song et al. 2003; Sun et al. 2017; Wang et al. 2016; Wen et al. 2016). It participates in tumor cell proliferation, migration, and invasion via multiple signaling pathways, including but not limited to PI3K/AKT, MAPK, and NF-κB (Wang et al. 2011). Therefore, MIIP may be a promising molecular target in ovarian cancer therapy.

In recent years, notable advancements and breakthroughs have emerged in the field of cancer treatment, attracting considerable attention to the biological therapeutic strategy of utilizing bacteria for targeted tumor therapy (Huang et al. 2021). The fundamental principle of bacterial therapy lies in the utilization of specific bacterial strains, such as oncolytic bacteria (*Salmonella typhimurium*, *Listeria monocytogenes*, *Clostridium novyi*, etc.), introduced into tumor tissues to exert anti-tumor effects (Y. Chen et al. 2021a, b; Wood and Paterson 2014). These bacteria, through deliberate design, possess the ability to selectively locate and infect cancer cells while exerting lesser influence on normal cells (Chowdhury et al. 2019). Among them, *S. typhimurium* (a facultative anaerobic bacterium) has gained widespread application in cancer research owing to its high tumor-targeting specificity, strong tumor tissue penetration capability, broad-spectrum anti-cancer effects, and favorable safety profile (Chen et al. 2022; Hamada et al. 2021; Liang et al. 2021; Mónaco et al. 2022; Wang et al. 2021). The attenuated strain of *S. typhimurium*, VNP20009, has been genetically modified by mutating the essential *purI* and *msbB* genes responsible for adenine synthesis and lipid A production, respectively (Clairmont et al. 2000). This modification enhances the colonization of the bacteria within hypoxic tumor regions while reducing its toxicity to the host organism (Felgner et al. 2017). The efficacy of this modified strain has been validated in numerous preclinical and Phase I clinical trials (Heimann and Rosenberg 2003; Toso et al. 2002). Our preliminary work has also demonstrated that engineered bacteria VNP20009, designed to express apoptosis-inducing factor (AIF) and programmed cell death protein 1 (PD-1), successfully inhibits the progression of melanoma in mice (Wang et al. 2020; Zhou et al. 2022). Based on these findings, we hypothesize that VNP20009, as a targeted oncolytic bacterium, and MIIP, as an anti-cancer protein, may synergistically enhance the anti-tumor effect, potentially addressing the current shortcomings of drug therapy.

In this study, the engineered strain VNP20009-AbVec-Igκ-MIIP was first constructed, and its characteristics were validated in vitro. Subsequent animal experiments were conducted to explore its potential anti-tumor effects on mouse subcutaneous tumors and investigate its mechanisms of action. The research aims to confirm the anti-tumor effects exerted by VNP20009-AbVec-Igκ-MIIP and to explore new breakthroughs for the challenges faced by current ovarian cancer drug treatment, such as drug resistance, low specificity, and side effects.

## Materials and methods

### Construction and in vitro evaluation of engineered strains

#### Construction of engineered bacteria

The MIIP gene (NCBI Reference Sequence: NM _ 021933.4) was synthesized by GENEWIZ Company (Suzhou, China). It was then incorporated into the eukaryotic expression vector AbVec-Igκ, which was provided by FitGene Biotechnology Company (Guangzhou, China). The resulting recombinant plasmid, named AbVec-Igκ-MIIP, carries Ampicillin (Amp) resistance. Subsequently, the successful construction of this plasmid was confirmed through agarose gel electrophoresis, followed by plasmid transformation experiments.

Two microliters of the recombinant plasmid (concentration not less than 500 ng/μL) was gently mixed with 50 μL of ice-cold competent VNP20009 (ATCC 202165) suspension. The mixture was then added to a pre-chilled electroporation cuvette with a 0.1-cm gap on ice, allowed to stand for 5 min, and subjected to electroporation under the conditions of 1800 V, 200 Ω, and 25 μF using a BIO-RAD Micropulse electroporator. After electroporation, the bacterial solution was promptly transferred to SOC medium and incubated with shaking at 37 °C for 1.5 h to obtain the engineered VNP20009-AbVec-Igκ-MIIP strain.

#### Bacterial growth curve determination

The turbidity method was employed to determine the bacterial growth curve. As the concentration of bacterial suspension is directly proportional to its turbidity, the optical density of the bacterial suspension could be measured using a spectrophotometer to infer the concentration of the culture. Activated bacterial solutions, VNP20009 (V), and VNP20009-AbVec-Igκ-MIIP (VM) were each inoculated with 1 mL into 100 mL of LB medium, then cultured at 37 °C with 150 rpm. Bacterial suspension optical density was measured at a wavelength of 600 nm at 0, 2, 4, 6, 8, 10, 12, 14, 16, 18, 20, 22, and 24 h. Growth curves were plotted with time on the x-axis and optical density values on the y-axis. The experiment was repeated three times.

#### Stability testing of engineered bacteria

The stability of plasmids carrying the Amp resistance gene in bacteria was determined through antibiotic screening. Bacteria that lose the plasmid during successive passages will be unable to survive in Amp-containing culture media. The VM strain was inoculated overnight in LB liquid medium without antibiotics, followed by continuous subculturing for 30 days in LB medium containing 50 μg/mL Amp. Plate counts were performed every 3 days. The stability of the plasmid was defined as the ratio between the bacterial count on each third day to the bacterial count on the first day.

### Ovarian cancer cell culture

ID8 (RRID: CVCL_IU14) cells were sourced from Fuxiang Biological Company in Shanghai, China, and cultured in DMEM medium (Gibco, CA, USA). Incubation was performed at 37 °C with a 5% CO_2_ atmosphere until the cells reached the logarithmic growth phase. Subsequently, the cells were harvested and suspended in PBS to prepare a suspension for subsequent experiments.

### Bacteria and cell co-culture experiments

#### Ability of live bacterial count method to detect bacterial entry into cells

The colony counting method was utilized to determine the count of viable bacteria to assess the strain’s capacity to infiltrate cells and its cytotoxic potential. In a six-well plate, 1 × 10^6^ ID8 were placed cells per well and allowed them to reach confluence while being cultured in DMEM medium. Subsequently, V and VM strains were co-cultured with ID8 cells for 2 h, maintaining a bacterial-to-ID8 cell ratio of 100:1. After co-culture, three PBS washes were performed to eliminate bacteria that had not invaded the ID8 cells. Then, DMEM medium supplemented with 1% penicillin/streptomycin was introduced, and the cultivation was continued for durations of 3, 6, and 10 h, respectively. Cells were collected at these three time points, lysed with PBS containing 0.1% Triton-X100, and the lysates were diluted and cultured on LB solid medium for counting, assessing bacterial survival after 3, 6, and 10 h of antibiotic treatment. The experiment was repeated three times.

#### Cell scratch assay

In a six-well plate, ID8 cells were seeded at a concentration of 5 × 10^6^ cells per well. Bacteria were introduced into the culture when the cells had reached confluence as a monolayer. The V and VM strains were introduced into separate wells at a bacteria-to-cell ratio of 100:1 and co-incubated for 2 h. The cells were washed three times with PBS to eliminate bacteria that did not invade the ID8 cells. A gentle scratch was created in each well using a 200-μL pipette tip, and meticulous observation and documentation were carried out under a microscope. This moment was marked as 0 h. The scratch marks were recorded again at 12 and 24 h, respectively. Each group selected five fields of view to take pictures, and the experiment was repeated three times.

#### Cell proliferation assay using CCK-8

The prepared cell suspension was inoculated into 96-well plates, approximately 100 μL per well. Subsequently, the culture plate was placed into an incubator for pre-cultivation (37 ℃, 5% CO_2_) until the cells adhered to the well surface. Following cell adhesion, they were divided into ID8 group (without bacteria), ID8 + V group (with 10^5 CFU/100 μL of V), and ID8 + VM group (with 10^5 CFU/100 μL of VM). Following co-cultivation with bacterial cells for 2 h, the bacteria that did not invade the cells were washed off. The plate was placed back into the incubator and incubated for 6, 12, 24, and 48 h. Afterward, 10 μL of CCK-8 solution was added to each well, and gentle shaking was applied to ensure thorough mixing. The plate was then placed in a CO_2_ incubator for an additional incubation period of 1–4 h. Subsequently, a microplate reader was employed to measure the absorbance at 450 nm. Each group was composed of five replicate wells, and the experiment was repeated thrice.

### Construction of mouse model and in vivo intervention

This research involving animal experimentation was granted approval by the Ethics Committee for Experimental Animals of Nanchang LeYou BioTech Co., Ltd., China (Approval ID: RYE2021112901).

Forty-eight female C57BL/6 wild-type mice aged 6 to 8 weeks were purchased, with a weight range of 18–20 g. All mice were kept in an SPF facility, with free access to both nourishment and hydration. After a period of 1 week for acclimatization, all groups of mice except for the control group underwent subcutaneous injection on the right flank with 100 μL of 5 × 10^6^ ID8 cells, to establish the tumor model. Modeling success was confirmed when the average tumor volume reached a range of 25 ~ 40 mm^3^. Following successful tumor modeling, the mice were randomly grouped, with 12 mice in each group: Group C (blank control group, administered 100 μL of PBS by gavage), Group M (ovarian cancer group, administered 100 μL of PBS by gavage), Group V (treatment group, administered V (1 × 10^7^ CFU/100 μL) by gavage), and Group VM (treatment group, administered VM (1 × 10^7^ CFU/100 μL) by gavage). The oral gavage frequency for each group was conducted once every 3 days.

On the 56th day, humane euthanasia was conducted on the mice, and tumor tissues were harvested. Some of the collected tissues were preserved by fixation in a 4% paraformaldehyde solution, while the remaining samples were cryopreserved at a temperature of − 80 °C. The calculation of tumor volume was performed utilizing the formula: volume = length × width^2^ × 0.5.

### Detection of bacteria in tissues

Blood, liver, tumor, and other tissue samples were collected from groups V and VM mice at 24 and 72 h after the initial oral gavage, with three mice per group at each time point. The collected samples were weighed and subsequently thoroughly homogenized under sterile conditions. Later, a suspension was prepared using 1 mL of sterile PBS, followed by serial dilution. Next, 10 μL of the supernatant was spotted onto both Amp-free LB solid media and LB solid media containing Amp. Then, they were incubated at 37 °C, and colony counting was conducted after 24 h.

### H&E staining and immunohistochemistry

Mouse tumor tissues were first collected and then immersed in a 4% paraformaldehyde solution for the purpose of fixation. After the fixation, the tissues were longitudinally sectioned into 5-μm-thick slices using a Leica CM1850 microtome (Leica, Germany). Following this, the slices were subjected to staining with hematoxylin and eosin. Then, they were subjected to morphological feature analysis using an optical microscope (Nikon Eclipse 80i, Nikon, Japan) equipped with NIS-Elements 3.2 software.

The tissue sections were subjected to deparaffinization in xylene, followed by rehydration in ethanol of varying concentrations. Subsequently, the sections were rinsed with PBS four times, with each rinse lasting 6 min. To prevent non-specific binding, a 3% horse serum incubation was performed on the sections. The sections were then incubated overnight at 4 °C with the primary antibodies, including the EGFR antibody (GB111504-100, Servicebio, China) and the MIIP antibody (20630–1-AP, Proteintech, USA). Subsequently, after the addition of secondary antibodies, chromogenic substrate, dehydration, and sealing, the slides were scanned using the VS120 microscope (Olympus Corporation, Tokyo, Japan), and the images were collected. Five images are randomly selected for each group. Quantitative analysis of these images was performed using ImageJ software (NIH, Bethesda, MD, USA) (Liu et al. 2022).

### Western blot

The extraction and release of proteins from murine tumors were conducted utilizing a lysis buffer (composed of RIPA lysis solution with a PMSF ratio of 100:1) at a temperature of 4 °C. Following centrifugation, the resultant supernatant was gathered. The determination of protein concentration was accomplished through the BCA assay. Following this, a suitable quantity of protein samples underwent electrophoresis on a 10% SDS–polyacrylamide gel, followed by their transfer onto a PVDF membrane (Millipore). At room temperature, the membrane was blocked for 2 h and then incubated overnight at 4 °C with specific primary antibodies, including: MIIP (20,630–1-AP, Proteintech, USA), β-actin (3700 s, CST, USA), Occludin (66,387–1-Ig, Proteintech, USA), PI3K (ab151549, Abcam, UK), p-PI3K (#17366 s, CST, USA), AKT (#9272 s, CST, USA), p-AKT (#4060 s, CST, USA), Bcl-2 (3498 s, CST, USA), Bax (2772 s, CST, USA), Ras (3965 s, CST, USA), MEK1/2 (4694 s, CST, USA), p-MEK1/2 (9154 s, CST, USA), ERK1/2 (ab184699, Abcam, UK), and p-MEK1/2 (AF1015, Affinity, USA). Afterward, the membrane was incubated with secondary antibodies, either anti-mouse or anti-rabbit, for 2 h. Lastly, following treatment with a chemiluminescent solution (Thermo Fisher, 32,209), these particular proteins were identified and captured using a chemiluminescent detection system (Tanon-5200). The target bands were quantitatively analyzed using Image J software.

### Fecal microbial DNA extraction and qPCR

Mouse fecal DNA was extracted using the MolPure ® Stool DNA Kit (Yeasen, Shanghai, China). The quantitative PCR amplification of the extracted fecal DNA was conducted utilizing the ViiA7 PCR System (Applied Biosystems, USA) with the employment of the Universal Blue qPCR SYBR Green Master Mix kit (Yeasen, Shanghai, China). The reaction system consisted of 10 μL MIX, 0.4 μL Forward Primer (10 μM), 0.4 μL Reverse Primer (10 μM), template DNA, and sterile ultrapure water to achieve a final volume of 20 μL. The amplification protocol commenced with an initial denaturation step at 95 °C, lasting 30 s. Subsequently, it underwent 40 cycles of denaturation at 95 °C for 3 s each, followed by annealing and extension at 60 °C for 20 s each cycle. Subsequently, data analysis was carried out using the 2^−ΔΔCT^ methodology (Livak and Schmittgen 2001). Reference details of the primer sequences utilized in this assay can be found in Supplementary Table S1.

### Data and statistical analysis

GraphPadPrism software (version 9.5.1, CA, USA) was utilized for the analysis of all the employed data. Two-tailed Student’s *t* test, one-way analysis of variance (ANOVA), and repeated measures analysis of variance (ANOVA) were used to evaluate statistical differences. All experimental outcome data were presented in the format of mean ± standard deviation (SD). Statistical significance was considered at *p* < 0.05, with “*” indicating *p* < 0.05 and “**” indicating *p* < 0.01. “Ns” indicates no statistical significance.

## Results

### Construction and in vitro evaluation of the engineered VM

**Fig. 1:**
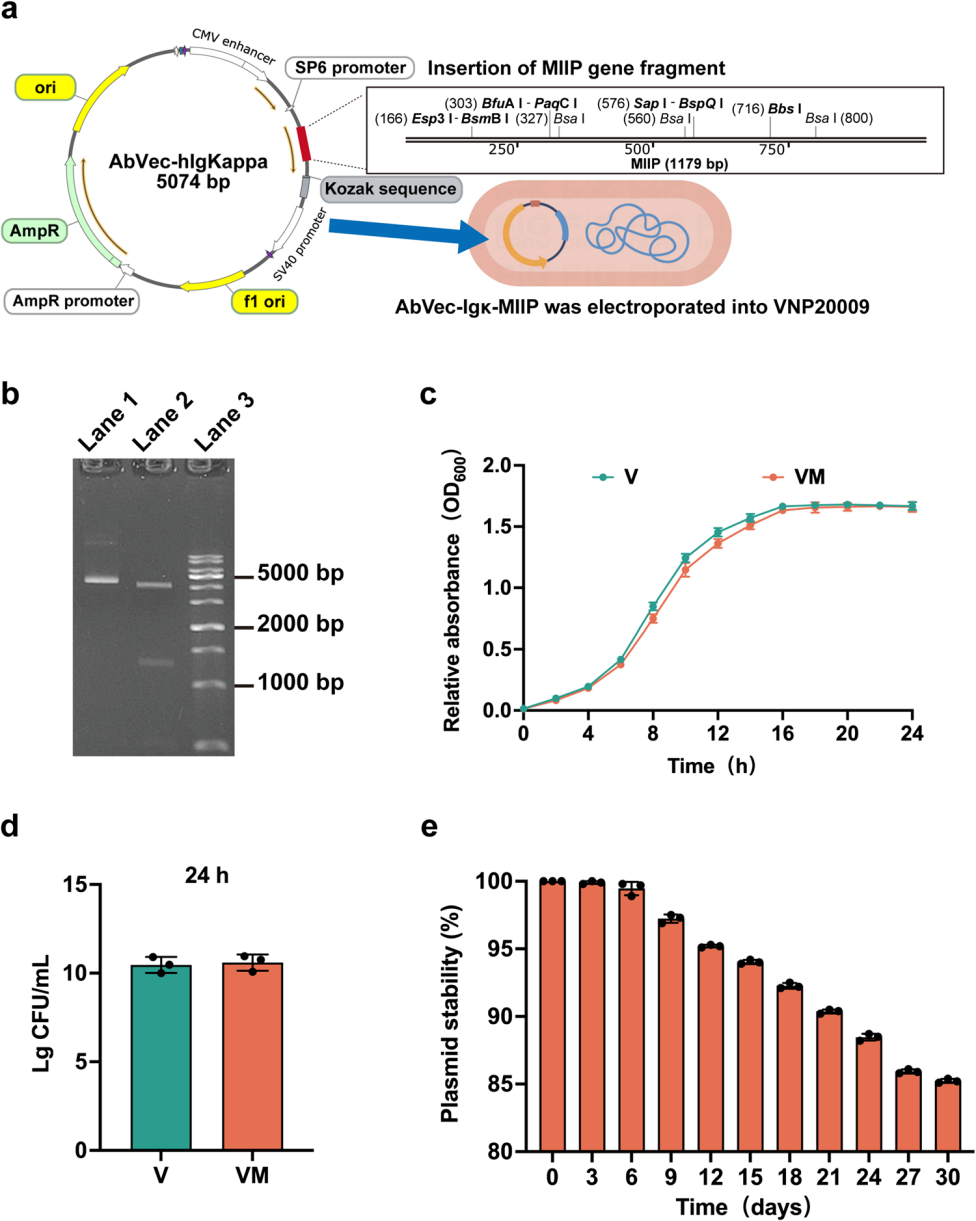
Construction and in vitro evaluation of VM-engineered strain. **a** Construction of engineered strains. **b** Agarose gel electrophoresis. Lane 1: Recombinant plasmid (undigested), Lane 2: Recombinant plasmid (digested with Apal I), Lane 3: 1 kb DNA Ladder. **c** Growth curves of V and VM. **d** Bacterial numbers of V and VM after 24 h of growth. **e** Plasmid stability test of VM. Statistical analysis involved a two-tailed *t* test (**c**, **d**) and one-way ANOVA followed by post hoc multiple comparison test (**e**)

Initially, we introduced the plasmid carrying the MIIP gene fragment into the VNP20009 strain via electroporation, resulting in the successful construction of the VM strain (Fig. 1a). Subsequently, we confirmed the presence of the MIIP band at the 1179 bp position following enzymatic digestion of the recombinant plasmid using agarose gel electrophoresis (Fig. 1b, lane 2). The growth kinetics of both V and VM strains were monitored within a 24-h period using the nephelometric method. Both V and VM strains exhibited logarithmic growth after 4 h, followed by a stable growth phase after 16 h (Fig. 1c). There were no noticeable variations in growth characteristics observed between the V and VM strains. By the 24-h mark, the concentrations of strains V and VM had reached 10^10^ CFU/mL (Fig. 1d). Following this, we assessed the plasmid stability of VM, passaging it daily for a consecutive period of 30 days. The plasmid stability reached 85% (Fig. 1e). These findings indicate that the engineered bacterium VM exhibits favorable in vitro growth characteristics.

### VM hinders ovarian cancer cell proliferation and migration

**Fig. 2:**
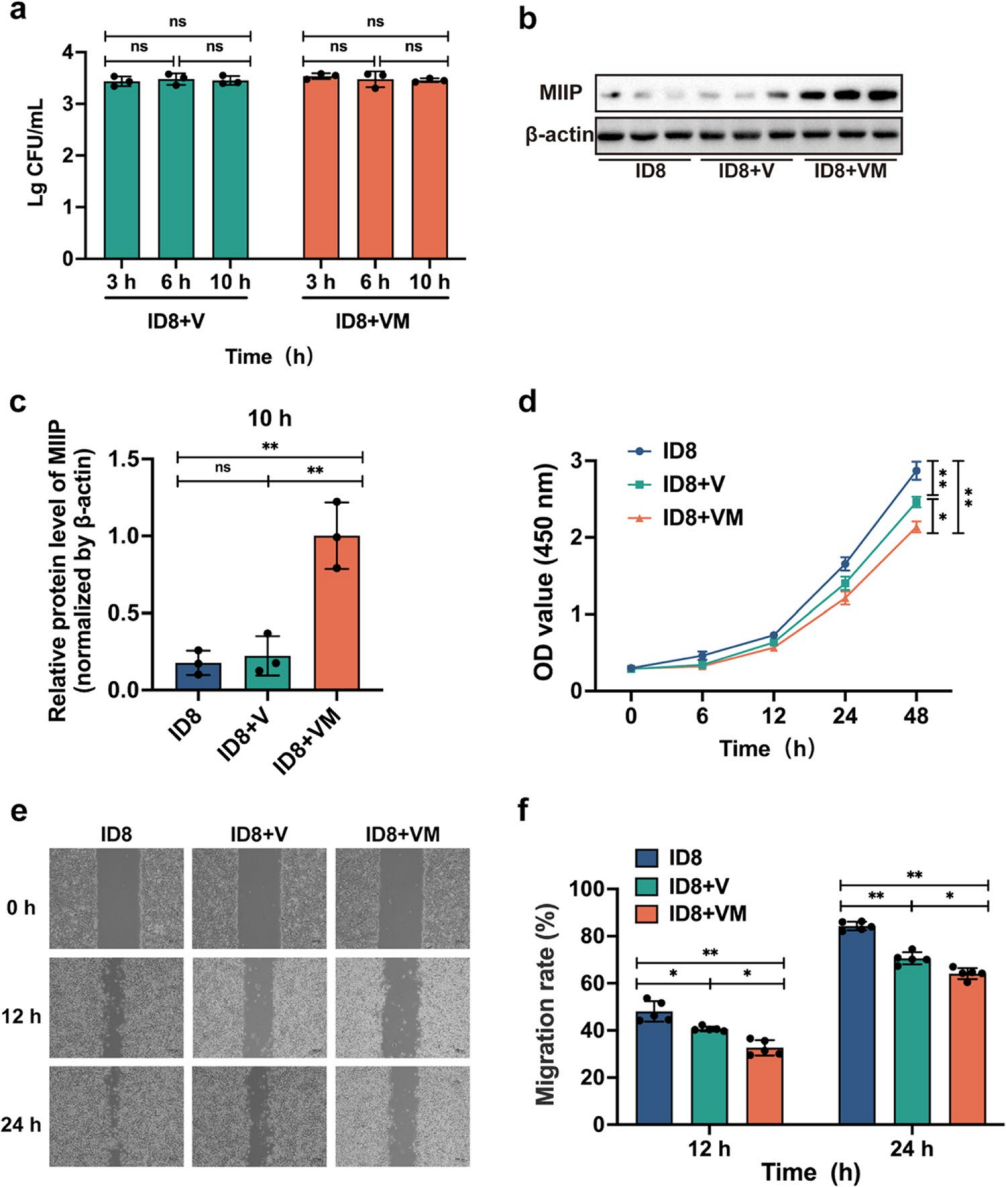
VM inhibits the proliferation and migration of ID8 cells in vitro. **a** At the 3-h, 6-h, and 10-h time points, the quantity of bacteria was assessed in the ID8, ID8 + V, and ID8 + VM groups. **b** At the 10-h time point, the expression levels of MIIP protein were determined by western blot. **c** ImageJ was used to quantify the expression levels of MIIP and β-actin, and the MIIP-to-β-actin ratio was calculated. **d** Cell proliferation assay analyzed by CCK8: The cell OD values were detected at 6, 12, 24, and 48 h, respectively. **e** Cell migration determined by scratch test. **f** The area of scratch regions was quantified using ImageJ, and the cell migration rate was calculated. Statistical analysis involved a one-way ANOVA and Tukey’s multiple comparison test (**a**, **c**, and **f**). OD value of cells at different time points is analyzed by repeated-measurement ANOVA and post hoc multiple comparison test (**d**). Data are represented as mean ± SD. * *p* < 0.05, ** p < 0.01

Next, employing the live bacterial counting method, the enumeration of bacteria that infiltrated the cells was assessed. The results indicated that the quantities of the V and VM strains within the cells remained relatively constant at 3, 6, and 10 h post-invasion, maintaining concentrations within the range of 10^3^ to 10^4^ CFU/mL (Fig. 2a). Furthermore, validation via western blot experiments revealed a significant upregulation of MIIP expression by 4.5–5 times compared with the control group after 10 h of VM entering the cells, *p* < 0.01(Fig. 2b, c).

To evaluate the influence of bacteria on cellular growth, a CCK-8 assay was performed. It was observed that, with increasing time, cells supplemented with the V and VM strains exhibited a gradual attenuation in their proliferative capacity. Notably, at 48 h, the proliferation capacity significantly decreased by 14.1% and 25.5% (*p* < 0.01, *p* < 0.01), respectively, as compared to the control group (Fig. 2d). Scratch assays were further employed to assess the bacterial capacity to inhibit cell migration. In comparison to the other two groups, the VM group displayed the lowest cell migration rate at 12 and 24 h, with percentages of 32.6% and 64.1%, respectively (Fig. 2e, f). These results collectively indicate that both the V and VM bacterial strains are capable of intracellular entry and can inhibit cell proliferation and migration. Additionally, engineered strain VM, which expresses the MIIP protein, exhibits a more potent inhibitory capacity.

### VM inhibits tumor growth in ovarian cancer-bearing mice

**Fig. 3:**
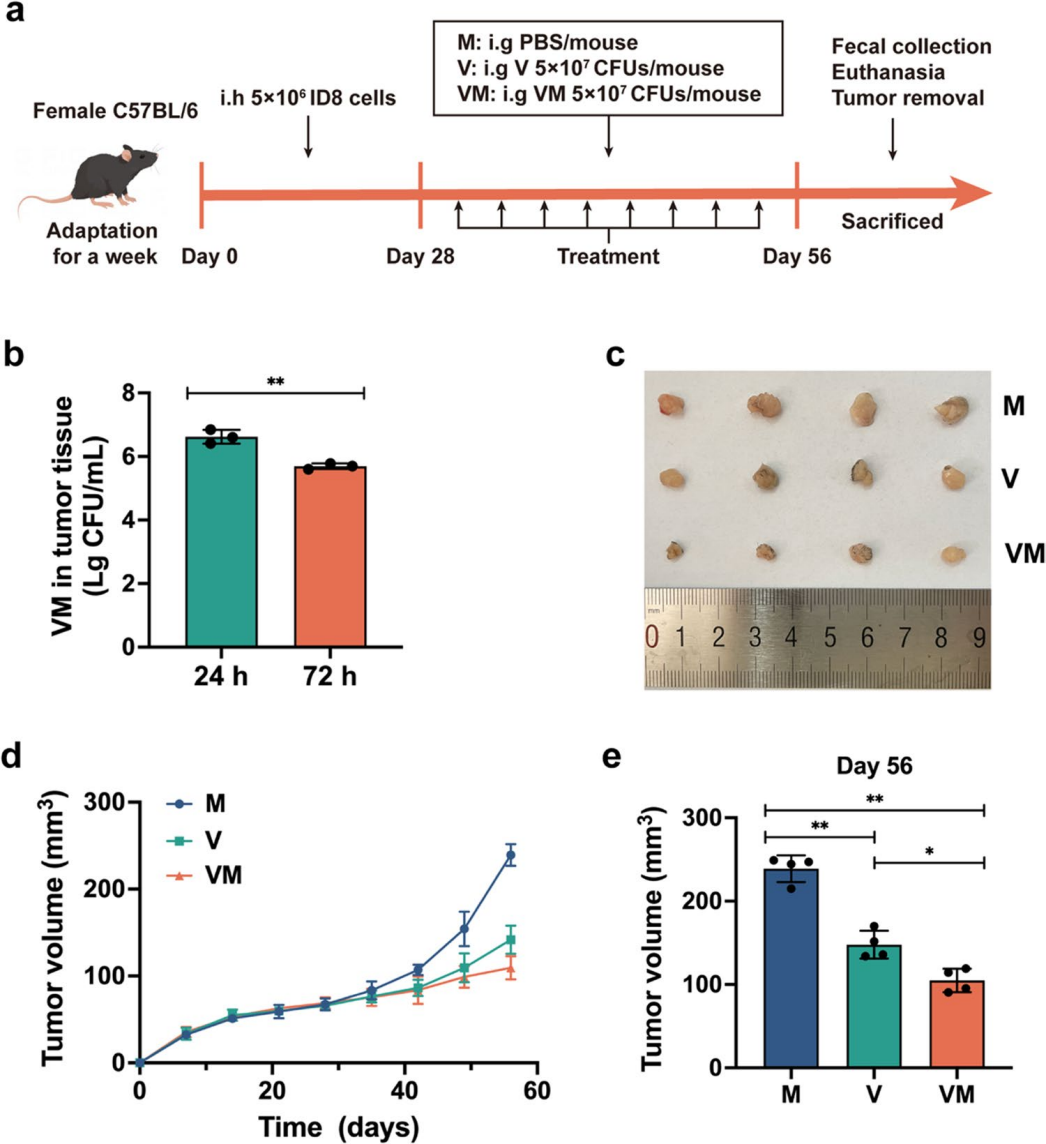
VM inhibits tumor growth in ovarian cancer-bearing mice. **a** Treatment schedule for ovarian cancer tumor-bearing mice. i.h.: subcutaneous injection; i.g.: administration by gavage. **b** VM gastric lavage for 24 and 72 h: the number of bacteria entering tumor tissue. **c** On day 56, images of tumors in the M, V, and VM groups were captured. **d** Alterations in tumor volume were monitored over a period of time in the M, V, and VM groups. **e** The tumor sizes of mice in each group were measured on day 56. Statistical analysis involved a two-tailed *t*-test (**b**) and one-way ANOVA followed by post hoc multiple comparison test (**d**, **e**). Data are represented as mean ± SD. Significance levels are represented as follows: * *p* < 0.05 and ** *p* < 0.01

To assess the anti-tumor properties of the genetically modified bacterium VM within the murine system, we established a xenograft mouse model of ovarian cancer (Fig. 3a). Initially, we conducted validation of VM’s capability to selectively home to tumor tissues, with the highest concentration reaching 10^6^ CFU/mL within 24 h (Fig. 3b). Subsequently, we diligently monitored the progression of tumor volume over time, revealing that both the V and VM groups exhibited substantial anti-tumorigenic effects commencing on day 35, markedly retarding tumor growth rates (Fig. 3d). On the 56th day, the tumor volumes in the VM group and V group decreased by 56.1% and 38.1%, respectively, compared to the M group (*p* < 0.01, *p* < 0.01). The VM group exhibited a more effective anti-tumor effect (Fig. 3e). These findings collectively indicate that engineered bacterium VM can selectively colonize murine tumor sites and inhibit tumor growth with enhanced effectiveness.

### VM suppresses the migratory and invasive abilities of tumor cells in ovarian cancer-bearing mice

**Fig. 4:**
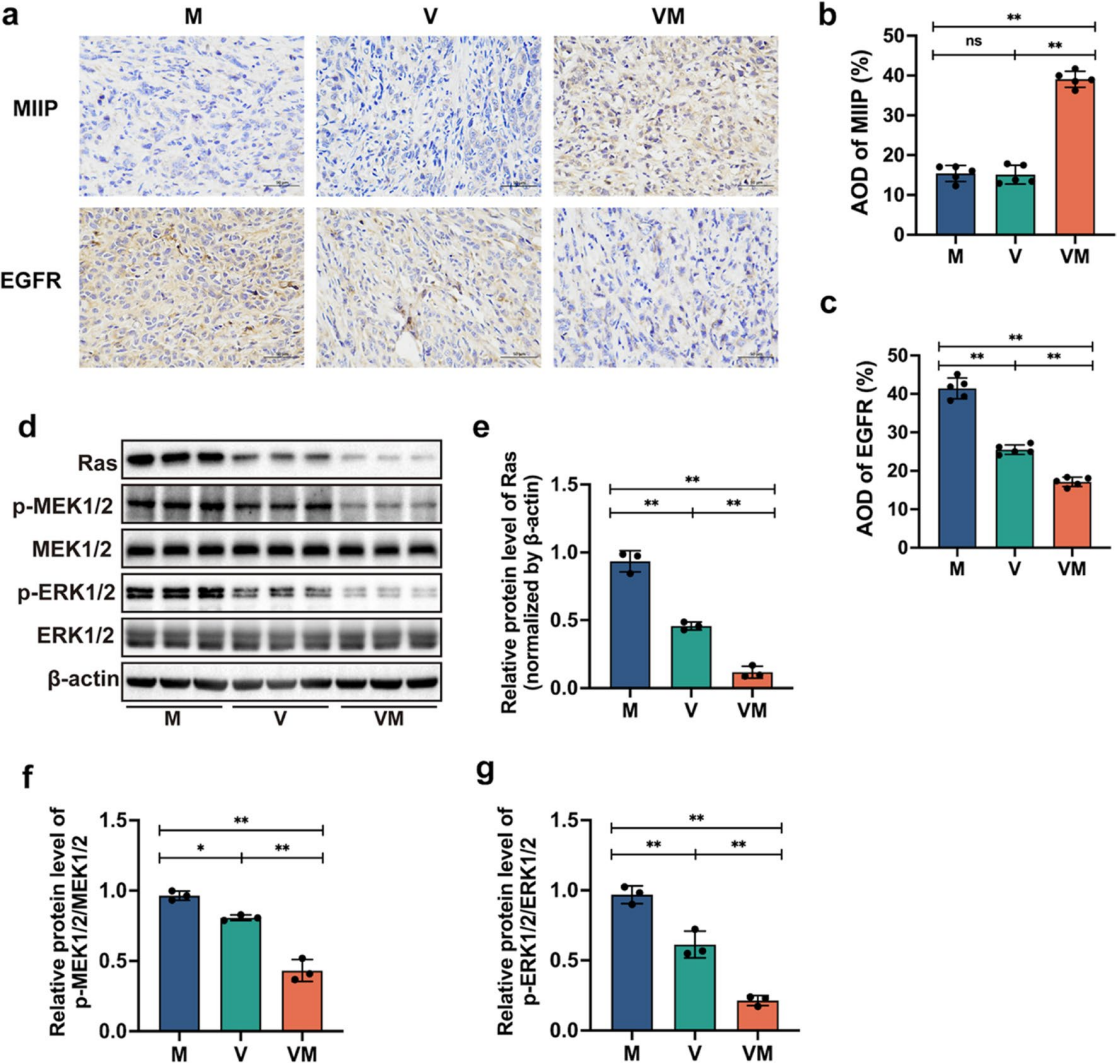
VM restrains the movement and infiltration of murine cancer cells. **a** Immunohistochemistry of MIIP and EGFR expression in tumor tissue (400 ×). **b** ImageJ analysis of the immunohistochemical positive area’s optical density value for the MIIP protein. **c** ImageJ analysis of the immunohistochemical positive area’s optical density value for the EGFR protein. **d** Protein expressions within the tumors were assessed using western blot analysis. (**e**) Ras, (**f)** p-MEK1/2/MEK1/2, and (**g**) p-ERK1/2/ERK1/2 were quantified using ImageJ to determine their relative levels. Statistical analysis involved one-way ANOVA followed by post hoc multiple comparison test (**b**, **c**, and **e**–**g**). Data are represented as mean ± SD. * *p* < 0.05 and ** *p* < 0.01

Considering that MIIP is regarded as an inhibitor of cellular migration and invasion, and it promotes the degradation of the EGFR protein (Chen et al. 2017), while the signaling transduction of EGFR is also linked to the invasion and metastasis of tumors (Morishige et al. 2008), we conducted immunohistochemical analysis to investigate further how VM exerts its antitumor effects. The results revealed a significantly larger MIIP protein-positive staining area in the VM group (39.1%) in comparison to both the V and M groups (15.4%, 15.1%). In contrast, the EGFR-positive staining areas in the V and VM groups (25.5%, 17.2%) were notably reduced compared to the M group (41.4%) (Fig. 4a–c). Subsequently, we assessed the relevant proteins in the EGFR-induced downstream kinase cascade involved in cell migration using western blot analysis. The results demonstrate that, in contrast to group M, group V shows a downregulation in the protein expression levels of Ras, p-MEK1/2/MEK1/2, and p-ERK1/2/ERK1/2 by 51.1%, 16.3%, and 36.7% (*p* < 0.01, *p* < 0.05, *p* < 0.01), respectively. The downregulation trend is more pronounced in the VM group, with respective reductions of 87.5%, 55.2%, and 78.0% (*p* < 0.01, *p* < 0.01, *p* < 0.01) (Fig. 4d–g). These outcomes indicate that VM can suppress the migratory and invasive properties of ovarian cancer cells by modulating the EGFR/Ras/MEK/ERK signaling pathway.

### VM inhibits tumor cell proliferation and induces tumor cell apoptosis in mice with ovarian cancer

**Fig. 5:**
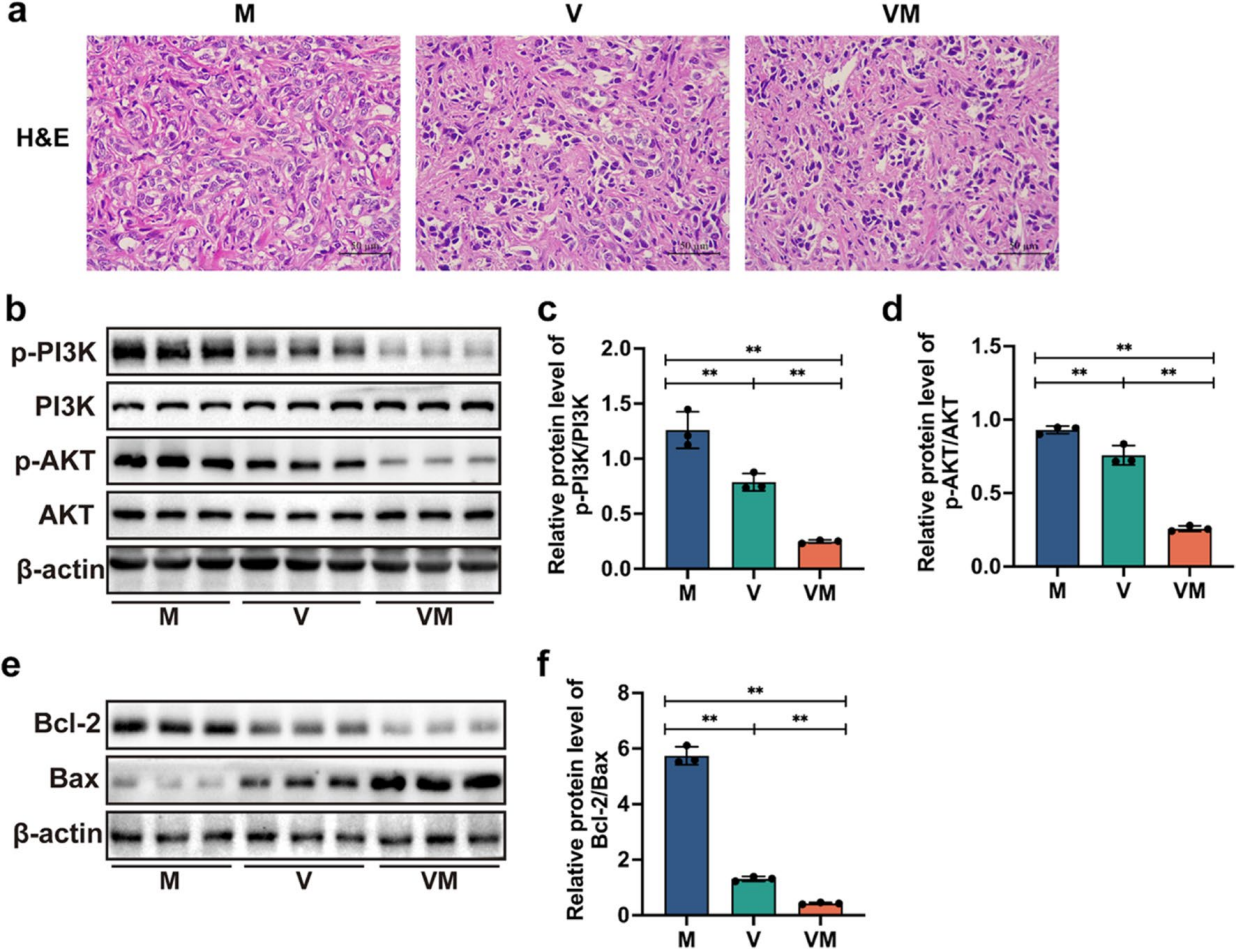
VM inhibits the proliferation of mouse tumor cells and induces apoptosis in tumor cells. **a** Photomicrographs showing tumor tissue specimens stained using the H&E method (magnification: 400 ×). **b** The expressions of proteins were assessed through western blot analysis on the tumor samples. The levels of (**c**) p-PI3K, PI3K, and (**d**) p-AKT, AKT were assessed using ImageJ for quantification. **e** Proteins were examined via western blot in tumor samples to assess their expressions. **f** The expression levels of bcl2 and bax were quantified through ImageJ, and their ratio was calculated. Statistical analysis involved one-way ANOVA and post hoc multiple comparison test (**c**, **d**, and **f**). Data are represented as mean ± SD. * *p* < 0.05 and ** *p* < 0.01

To assess the impact of VM administration on cellular proliferation and apoptosis, we conducted histological analysis using H&E staining. The results revealed that within the M group, there was a wide variety of morphological characteristics observed in tumor cells, along with an increased frequency of mitotic figures and disorganized cellular arrangements. Conversely, within the VM group, tumor cell morphologies tended to be more uniform, the frequency of mitotic figures decreased, cellular volume reduced, and nuclear condensation was observed (Fig. 5a). This indicates that the proliferation of tumor cells was inhibited, and apoptosis of cells occurred. Subsequently, the tumor proliferation signaling pathway-related protein expression was further analyzed via western blot. VM significantly reduced the expression of p-PI3K/PI3K and p-AKT/AKT, downregulating them by 80.4% and 72.2% (*p* < 0.01, *p* < 0.01), respectively, compared to the M group (Fig. 5b–d). Subsequently, the apoptotic pathways regulated by the Bcl-2 protein family were further detected. In comparison to the M group, the VM group exhibited a noteworthy 92.5% reduction in the Bcl-2/Bax expression ratio, *p* < 0.01(Fig. 5e, f). These findings underscore the suppressive impact of VM on tumor cell proliferation and its facilitative role in tumor cell apoptosis.

### The impact of VM intragastric administration on the intestinal microbiota of ovarian cancer mice

**Fig. 6:**
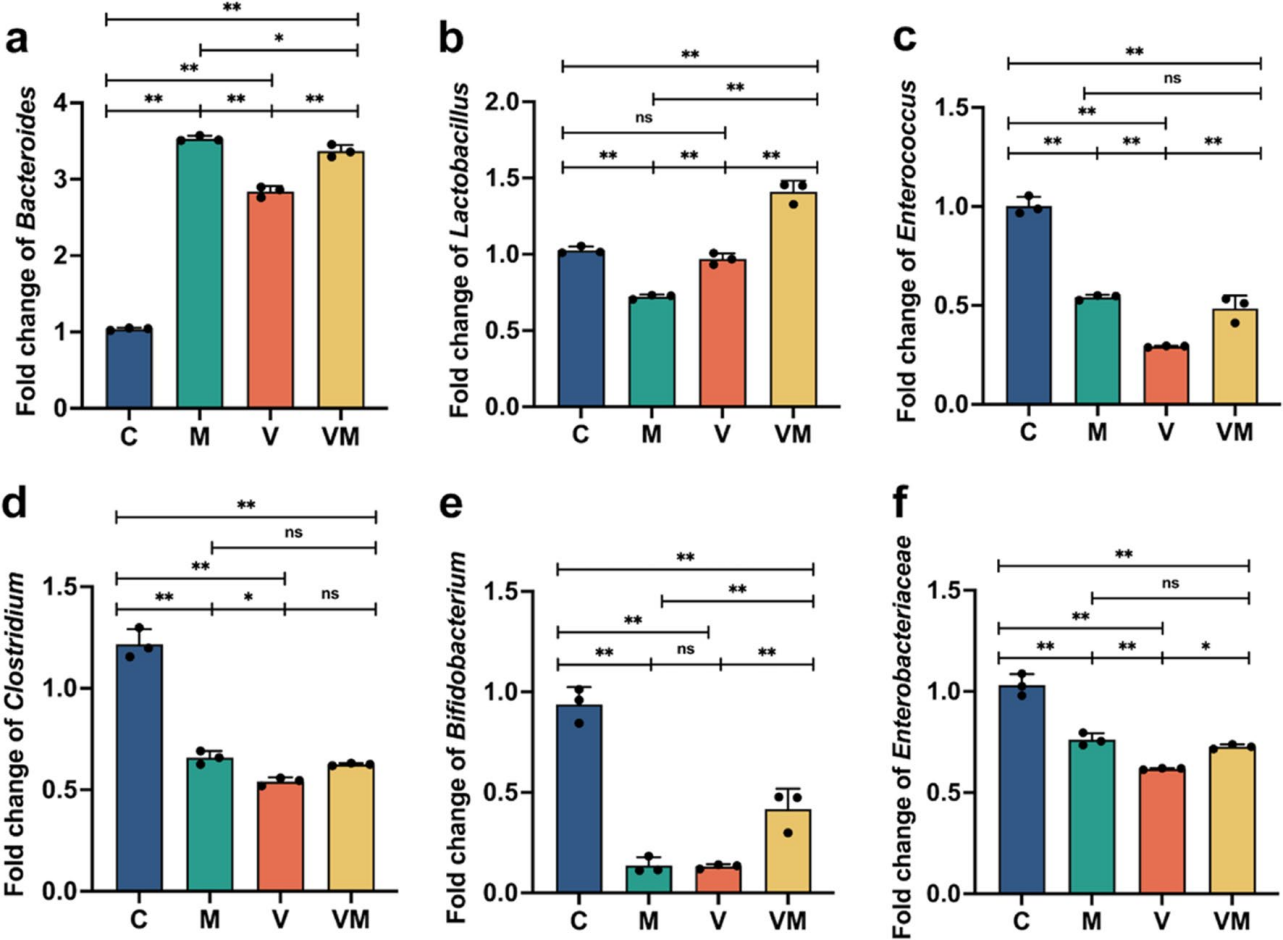
The effect of VM gavage on the gut microbiota of mice. Relative numbers of *Bacteroides*, *Lactobacillus*, *Enterococcus*, *Clostridium*, *Bifidobacterium*, and *Enterobacteriaceae* in mice intestines (**a**–**f**) using q-PCR. Statistical analysis involved one-way ANOVA and post hoc multiple comparison test (**a**–**f**). Data are represented as mean ± SD. * *p* < 0.05 and ** *p* < 0.01

Finally, given our chosen route of administration via intragastric gavage, in order to elucidate the impact of V and VM on the predominant intestinal microbiota of the host, we extracted murine fecal DNA and subsequently employed real-time fluorescent quantitative PCR to assess the relative DNA content of *Enterococcus*, *Enterobacteriaceae*, *Clostridium*, *Lactobacillus*, *Bacteroides*, and *Bifidobacterium*. The results show that, compared to Group C, Group M exhibited a 70.6% increase in the DNA content of *Bacteroides*, with a significance level of *p* < 0.01 (Fig. 6a). Additionally, the DNA content of *Lactobacillus*, *Enterococcus*, *Clostridium*, *Bifidobacterium*, and *Enterobacteriaceae* decreased by 29.7%, 46.0%, 45.9%, 85.4%, and 26.1%, respectively, in Group M compared to Group C, with all differences being statistically significant at *p* < 0.01 (Fig. 6b–f). This suggests that the predominant intestinal microbiota in ovarian cancer mice differs from that in normal mice. Furthermore, in comparison to the C group, the VM group displayed a consistent trend of increased or decreased DNA content for all bacteria, except for *Lactobacillus*, which exhibited incongruent changes compared to the M group. The above findings suggest that intragastric administration of V and VM may have a limited impact on the predominant intestinal microbiota in mice.

## Discussion

Ovarian cancer, as a common malignancy in gynecology, poses significant challenges during its treatment, including chemotherapy resistance, recurrence, and severe adverse reactions (Wen et al. 2021). In recent years, bacterial-based cancer therapy has emerged as a potential treatment method to overcome these challenges (Lin et al. 2021). This study delves into the anti-tumor potential of the genetically engineered strain VM, providing fresh perspectives and avenues for advancing clinical approaches in ovarian cancer therapy.

The VNP20009 we utilized in our research is a genetically modified bacterium. It exhibits specific chemotaxis and intrinsic anti-tumor properties in the hypoxic, nutrient-rich, and immune-suppressed tumor microenvironment (Lin et al. 2021). Consequently, it is employed as an active delivery carrier for various anti-cancer drugs, such as cytokines, cytotoxic agents, regulatory molecules, tumor-associated antigens or antibodies, prodrug enzymes, and siRNA (Liang et al. 2019). In a previous research report, VNP20009 carrying Sox2 shRNA constructs targeted lung cancer cells and silenced the Sox2 gene, effectively inhibiting tumor growth in heterotransplanted lung cancer mice (Zhao et al. 2016). In another study, VNP20009 was engineered to target tumor sites and deliver the tumor suppressor Tumstatin, promoting apoptosis in mouse melanoma cells and thereby suppressing tumor growth (Bao et al. 2022). Based on the findings from previous studies, robust support is provided for the design of VNP20009 as an engineered bacterium (VM) expressing the anticancer protein MIIIP and its application in the field of ovarian cancer therapy. Our results demonstrate that in vitro experiments confirm the outstanding growth performance and genetic stability of VM (Fig. 1). Co-cultivation of the VM strain with ovarian cancer cells has revealed the bacterium’s ability to invade cancer cells and stably express the MIIP protein, concomitant with inhibiting ovarian cancer cell proliferation and migration (Fig. 2). These performances of VM imply that it may become a potential therapeutic approach. Nevertheless, additional in vivo investigations are necessary to confirm the inhibitory effects of this engineered bacterium on solid tumors.

Subsequently, we established a murine ovarian cancer model to assess the inhibitory capability of VM against ovarian cancer in vivo. The results indicate that V and VM mainly accumulate in the tumor tissue (Fig. 3). This may reduce the impact on other tissues, suggesting that they may have the potential as drugs for cancer treatment with high specificity and low toxic side effects. VNP20009 itself possesses tumor-killing characteristics, significantly inhibiting tumor growth. However, the engineered bacteria VM expressing MIIP exhibits a more pronounced inhibitory effect on tumors. Despite both in vitro and in vivo experimental results demonstrating the anti-tumor effects of VM, further molecular biology experiments are still needed in our research to unveil its exact mechanism of action.

As a genetic engineering bacterium, VM exhibits unique and synergistic anti-tumor effects through its bacterial components VNP20009 and anti-cancer protein MIIP. At the bacterial level, the intrinsic anti-tumor mechanism of VNP20009 is not fully understood at present (Badie et al. 2021). It may potentially induce the death of tumor cells directly by promoting autophagy or apoptosis pathways (Li et al. 2021). Additionally, it could indirectly attack tumor cells by activating anti-tumor immune responses through its derived factors such as flagellar proteins, lipoproteins, and lipopolysaccharides (J. Chen et al. 2021a, b). As a regulator of migration and invasion, MIIP, functioning as a protein, actively engages in the remodeling of the tumor cell cytoskeleton, cellular adhesion, and modulation of signaling pathways (Gao et al. 2022). EGFR, belonging to the receptor tyrosine kinase family, exhibits a close association between its aberrant expression and activation, and the genesis, progression, invasion, and metastasis of tumors (Voldborg et al. 1997). Through its tyrosine kinase activity, EGFR can activate the Ras protein, thereby initiating the Ras/MEK/ERK signaling pathway, ultimately inducing cellular proliferation, migration, and invasion (Du et al. 2019). Research has indicated that MIIP has the capability to expedite the degradation of EGFR while concurrently suppressing the downstream Ras/MEK/ERK signaling cascade, consequently curbing the proliferation and metastasis of non-small cell lung cancer (Wen et al. 2016). In another study, it was found that aberrant activation of the EGFR/ERK signaling pathway can lead to the proliferation, survival, invasion, and drug resistance of ovarian cancer cells (Cui et al. 2018). Therefore, we speculate that the inhibitory effect of VM on ovarian cancer may be mediated through the EGFR/Ras/MEK/ERK signaling pathway. Here, our immunohistochemistry and western blot results indicate that VM enhances MIIP expression, significantly downregulates EGFR positivity, thereby reducing the activation of Ras protein. This leads to a decrease in the phosphorylation levels of downstream MEK/ERK, consequently inhibiting the migration and invasion of tumor cells (Fig. 4). V also downregulates protein expression along the signaling pathway, but its effect is not as pronounced as VM. This suggests that VNP20009 can directly inhibit tumor cells through this signaling pathway on its own, but this effect is further enhanced when engineered into bacteria.

Research has shown that the PI3K/AKT signaling pathway plays a crucial role in the pathogenesis of ovarian cancer (Ediriweera et al. 2019). Aberrant activation of this signaling pathway can increase the rate of cellular proliferation and inhibit apoptosis (programmed cell death), leading to uncontrolled growth of cancer cells (Xu et al. 2021). Bcl-2 functions as a downstream signaling molecule within the PI3K/AKT pathway, working collaboratively with Bax to regulate the progression of cell apoptosis (Zhou et al. 2016). Phosphorylation of PI3K and AKT facilitates Bcl-2 release while dampening Bax activity, leading to the suppression of apoptosis in tumor cells (Liu et al. 2016). In a recent study, it was found that the PI3K/AKT signaling pathway can be regulated by MIIP to inhibit the progression of prostate cancer (Yan et al. 2019). In order to further validate whether VM can also suppress ovarian cancer through this pathway, we conducted additional research. In our research, H&E staining results reveal that VM promotes tumor cell necrosis, nuclear condensation, and fragmentation. This suggests that the proliferation of tumor cells is inhibited, leading to the occurrence of cellular apoptosis (Fig. 5). Furthermore, western blot results indicate that VM reduces the expressions of p-PI3K and p-AKT in tumor tissues, along with a decreased ratio of Bcl-2 and Bax protein expressions. This further substantiates that VM has the ability to suppress the proliferation of tumor cells and induce apoptosis in tumor cells via the PI3K/AKT signaling pathways.

In addition, in our study, dissimilarities in predominant intestinal microbiota between normal mice and ovarian cancer-afflicted mice were observed (Fig. 6). This finding is consistent with previous research results. Further analysis also revealed that the trend of changes in the gut microbiota in cancer mice, whether administered or not administered with bacteria, was generally consistent, with the exception of *Lactobacillus* showing a distinct trend after bacterial administration. This result indicates that gastric lavage with VM has a relatively minor impact on the intestinal microbiota of mice. This may be attributed to the relatively short duration of VM oral treatment. Consequently, we are unable to delve deeper into the potential effects of changes in the intestinal microbial community on ovarian cancer. However, in the study conducted by Chambers et al. (2022), disruption of the gut microbiota is considered to promote the development of ovarian cancer and inhibit sensitivity to chemotherapy. Therefore, it is essential for future research to further investigate the precise impact of high-dose and long-term VM gavage on the gut microbiota, as well as the potential implications of alterations in the gut microbiota on the efficacy and safety of VM.

In summary, our research indicates that VM possesses the capability to suppress ovarian cancer, opening up novel avenues for the application of genetically engineered bacteria in the field of ovarian cancer treatment. However, it is imperative to recognize that merely exploring the short-term impacts of VM on tumors through the establishment of an ovarian cancer mouse model falls far short of comprehensive assessment. Long-term treatment effects, potential development of drug resistance, as well as the safety and effectiveness of transitioning from experimental research to clinical applications, among other factors, also need to be taken into consideration. In the future, it is necessary to conduct more animal experiments and cell studies to assess the administration concentration and duration of VM, as well as its toxic side effects on vital organs. Additionally, further molecular biology experiments are needed for specific mechanistic exploration to clarify VM’s cellular-level mechanisms of action. This will provide a more reliable scientific basis for its clinical application.

## Supplementary Information

Below is the link to the electronic supplementary material.

Supplementary file1 (PDF 98 KB)

## Data Availability

All data generated or analyzed during this study are included in this article. The data sets used and/or analyzed during the current study are available from the corresponding author on reasonable request.
