# Correction: VNP20009-Abvec-Igκ-MIIP suppresses ovarian cancer progression by modulating Ras/MEK/ERK signaling pathway

**DOI:** 10.1007/s00253-025-13593-0

**Published:** 2025-09-16

**Authors:** Qian Wang, Yuwen Tang, Ang Dai, Tiange Li, Yulin Pei, Zuo Zhang, Xinyue Hu, Tingtao Chen, Qi Chen

**Affiliations:** 1 Department of Obstetrics and Gynecology, The 2nd Affiliated Hospital, Jiangxi Medical College, Nanchang University, 1 Minde Road, Donghu District, Nanchang City, 330000 Jiangxi Province China; 2 National Engineering Research Center for Bioengineering Drugs and the Technologies, Institute of Translational Medicine, Jiangxi Medical College, Nanchang University, No. 1299, Xuefu Avenue, Honggutan District, Nanchang City, Jiangxi Province China

**Correction: Applied Microbiology and Biotechnology (2024) 108:218**

10.1007/s00253-024-13047-z

After the publication of this article, the following problem has come to our attention:

The image of the VM group in Fig. 5a was mistakenly taken from the same HE-stained section as the V group, resulting in partial duplication between the V and VM group images. The authors replaced the third figure in Fig. 5a with this accurate version. The corrected Fig. 5 is provided below. These changes do not affect the conclusions of this work. The authors sincerely apologize for any inconvenience caused.

**Fig. 5:**
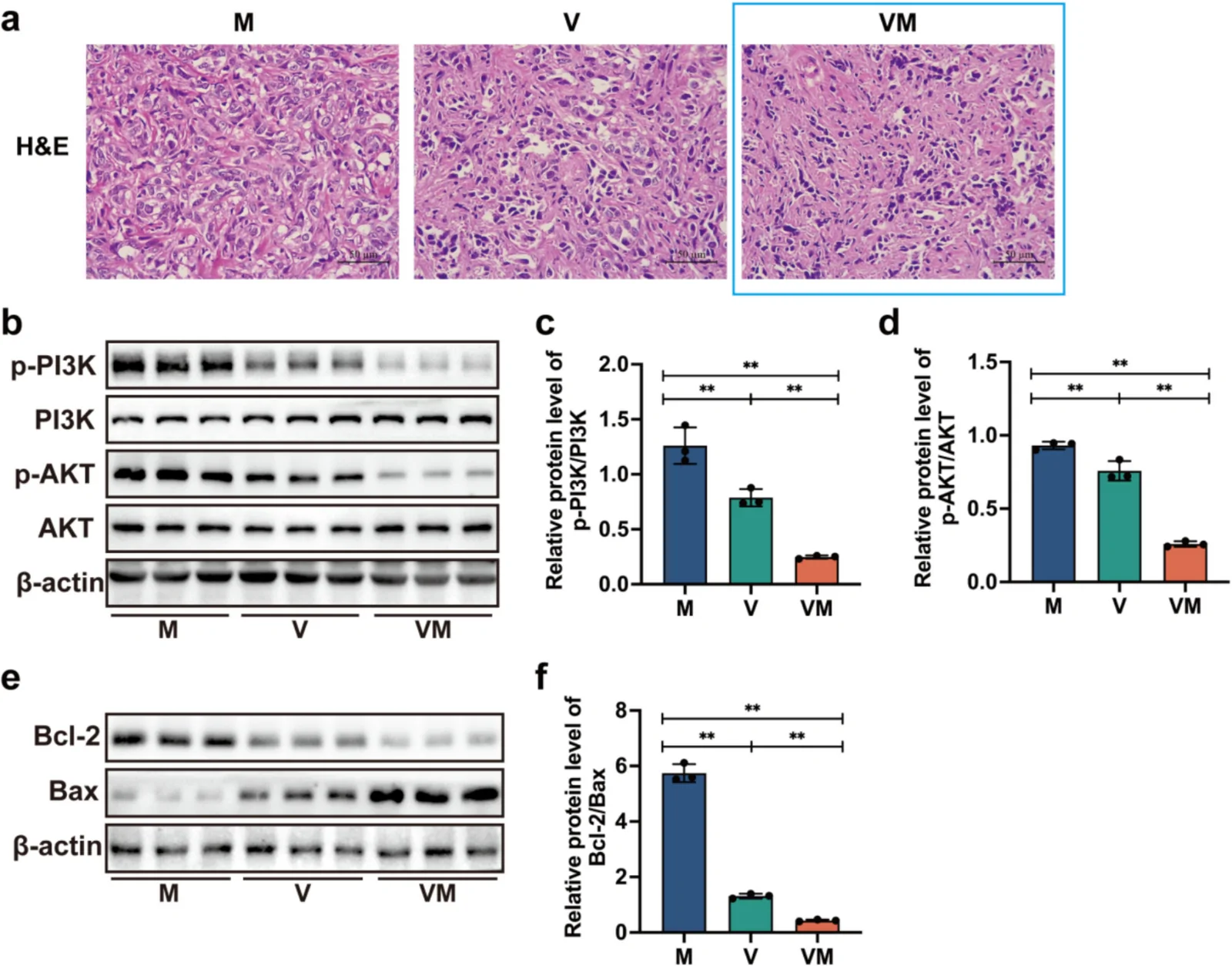
VM inhibits the proliferation of mouse tumor cells and induces apoptosis in tumor cells. **a** Photomicrographs showing tumor tissue specimens stained using the H&E method (magnification: 400×). **b** The expressions of proteins were assessed through Western blot analysis on the tumor samples. The levels of (**c**) p-PI3K, PI3K, and (**d**) p-AKT, AKT were assessed using ImageJ for quantification. **e** Proteins were examined via Western blot in tumor samples to assess their expressions. **f** The expression levels of Bcl-2 and Bax were quantifed through ImageJ, and their ratio was calculated. Statistical analysis involved one-way ANOVA and post hoc multiple comparison test (**c**, **d**, and **f**). Data are represented as mean ± SD. * *p* < 0.05, and ** *p* < 0.01

