# Supplementary material for: VNP20009-Abvec-Igκ-MIIP suppresses ovarian cancer progression by modulating Ras/MEK/ERK signaling pathway
Source: Appl Microbiol Biotechnol. 2024 Feb 19;108(1):218. doi: 10.1007/s00253-024-13047-z (PMC10876780; doi:10.1007/s00253-024-13047-z)
Supplement: Supplementary file 1 — Supplementary file1 (PDF 98 KB) [file 253_2024_13047_MOESM1_ESM.pdf]

Title Page

Journal: Applied Microbiology and Biotechnology

Manuscript Title: VNP20009-Abvec-Igκ-MIIP Suppresses Ovarian Cancer Progression by Modulating Ras/MEK/ERK Signaling Pathway

Authors' Names: Qian Wang[1], Yuwen Tang[1], Ang Dai[1], Tiange Li[1], Yulin Pei[1], Zuo Zhang[1], Xinyue Hu[1], Tingtao Chen[2*] & Qi Chen[1*]

1 Department of Obstetrics and Gynecology, The Second Affiliated Hospital of Nanchang University, Nanchang, China. Full postal address: 1 Minde Road, Donghu District, Nanchang City, Jiangxi Province, China.

2 National Engineering Research Center for Bioengineering Drugs and the Technologies, Institute of Translational Medicine, Nanchang University, Nanchang, China. Full postal address: No. 1299, Xuefu Avenue, Honggutan District, Nanchang City, Jiangxi Province, China.

* Correspondence to:

Prof. Dr. Tingtao Chen

E-mail: chentingtao1984@163.com

Phone:+8613979196862

Orcid: 0000-0002-0506-8536

Prof. Dr. Qi Chen

E-mail: chenqiyangbai@126.com

Phone: +8613807912933

Supplementary Material

Table S1. Primer sequences for qPCR

Primer	Forward (5’-3’)	Reverse (5’-3’)
Lactobacillus	TGGAAACAGRTGCTAATACCG	GTCCATTGTGGAAGATTCCC
Bifidobacterium	CTTACTTCGCCTTCTTTGCTCC RTAC	AGAAGTCCAAGACTTTGGCC CTGA
Enterococcus	GACGTTACCCGCAGAAGAAG	TACGCGCAGTAATTCCGAT
Enterobacteriaceae	GTTAATACCTTTGCTCATTGA	ACCAGGGTATCTAATCCTGTT
Bacteroides	CTGAACCAGCCAAGTAGCG	CCGCAAACTTTCACAACTGAC TTA
Clostridium	GCACAAGCAGTGGAGT	CTTCCTCCGTTTTGTCAA
16SrRNA	ACTCCTACGGGAGGCAGCAGT	TATTACCGCGGCTGCTGGC
